# Coping strategies and mental health outcomes of conflict-affected persons in the Republic of Georgia

**DOI:** 10.1017/S2045796016000019

**Published:** 2016-01-25

**Authors:** L. Saxon, N. Makhashvili, I. Chikovani, M. Seguin, M. McKee, V. Patel, J. Bisson, B. Roberts

**Affiliations:** 1 ECOHOST – The Centre for Health and Social Change, London School of Hygiene and Tropical Medicine, London, UK; 2 Global Initiative on Psychiatry, Tbilisi, Georgia; 3 Ilia State University, Tbilisi, Georgia; 4 Curatio International Foundation, Tbilisi, Georgia; 5 Centre for Global Mental Health, London School of Hygiene and Tropical Medicine, London, UK; 6 Cardiff University School of Medicine, Cardiff and Vale University Health Board, Cardiff, UK

**Keywords:** Conflict, coping, mental health, trauma

## Abstract

**Aims.:**

Adults who experienced the 1992 and 2008 armed conflicts in the Republic of Georgia were exposed to multiple traumatic events and stressors over many years. The aim was to investigate what coping strategies are used by conflict-affected persons in Georgia and their association with mental disorders.

**Method.:**

A cross-sectional survey was conducted with 3600 adults, representing internally displaced persons (IDPs) from conflicts in the 1990s (*n* = 1200) and 2008 (*n* = 1200) and former IDPs who returned to their homes after the 2008 conflict (*n* = 1200). Post-traumatic stress disorder, depression, anxiety and coping strategies were measured using the Trauma Screening Questionnaire, Patient Health Questionnaire-9, Generalised Anxiety and adapted version of the Brief Coping Inventory, respectively. Descriptive and multivariate regression analyses were used.

**Results.:**

Coping strategies such as use of humour, emotional support, active coping, acceptance and religion were significantly associated with better mental health outcomes. Coping strategies of behavioural and mental disengagement, denial, venting emotions, substance abuse and gambling were significantly associated with poorer mental health outcomes. The reported use of coping strategies varied significantly between men and women for 8 of the 15 strategies addressed.

**Conclusions.:**

Many conflict-affected persons in Georgia are still suffering mental health problems years after the conflicts. A number of specific coping strategies appear to be associated with better mental health and should be encouraged and supported where possible.

## Introduction

Populations affected by armed conflict are frequently exposed to traumatic events and daily stressors and are at a greater risk of elevated levels of mental health disorders (Miller & Rasmussen, 2009; Steel *et al.* 2009). A substantial body of research has examined risk-factors for poor mental health among conflict-affected civilian populations (Porter & Haslam, 2005; Steel *et al.* 2009), but fewer studies have examined protective factors. As a result, research on protective factors, including coping, has been identified as a priority research area for mental health and psychosocial support among conflict-affected populations (Tol *et al.* 2011).

Coping can be described as ‘an attempt to master, tolerate, or reduce internal or external stressors that an individual perceives as exceeding existing resources‘ (Folkman & Lazarus, 1980, 1991). Most current coping measures build upon the problem- and emotion-focused domains are suggested by Folkman & Lazarus (1980). If a situation is appraised as intractable or impossible to change, a person will employ emotion-focused modes of coping. Problem-focused modes are used when a person appraises a troubling situation as surmountable through action. Problem-focused coping entails an ‘attempt to solve, reconceptualise, or minimise the effects of a stressful situation,’ while emotion-focused coping includes ‘self-preoccupation, fantasy, or other conscious activities related to affect regulation’ (Parker & Endler, 1996).

Harnessing coping strategies which support good mental health may help reduce individuals needing more specialist mental health services (which are often not available in many conflict-affected and resource-poor settings). However, evidence is limited on the relationship between coping and mental health among conflict-affected populations in low- and middle-income settings (Seguin & Roberts, 2015) where the vast majority of conflict-affected populations live. The available evidence does suggest differences in coping strategies between men and women (Seguin & Roberts, 2015).

The Republic of Georgia has experienced two main phases of armed conflict in recent years, each involving secessionist movements. The first was in the 1990s, involving separatist movements in South Ossetia and Abkhazia that resulted in 300 000 people being forcibly displaced from their homes, of whom approximately 229 000 remain internally displaced persons (IDPs) (Ministry of Internally Displaced Persons from the Occupied Territories, 2014). The second phase followed the 2008 conflict between Georgia and the Russian Federation over South Ossetia, which led to 128 000 additional IDPs from South Ossetia, of whom around 30 000 remain displaced (Ministry of Internally Displaced Persons from the Occupied Territories, 2014).

The majority of IDPs currently live in government-established IDP settlements, with some remaining in makeshift settlements in former hotels, schools, factories and hospitals (particularly those displaced from the 1990s conflicts) (IDMC, 2014). Georgian IDP communities are characterised by poor living conditions, high unemployment, poverty and low access to health care (WHO/Children of Georgia, 2009). Their exposure to conflict-related violence, forced displacement and loss of loved ones, homes and livelihoods has been associated with prolonged poor mental health (Makhashvili *et al.* 2014; Roberts *et al.* 2014; Comellas *et al.* 2015). These losses are compounded by the strong attachment to their lost villages and farm lands and a feeling of betrayal by the failure to allow their return (Makhashvili *et al.* 2010). Many of the IDPs from the 2008 conflict who have now returned (returnees) to their home villages in the border region with South Ossetia also experience poor living conditions and economic prospects, limited access to basic services and amenities, including health care, and vulnerability to future violence (United Nations Security Council, 2009). Limited access to mental health services has also been reported among conflict-affected persons in Georgia (Chikovani *et al.* 2015). The objective was to investigate what coping strategies are used by conflict-affected persons in Georgia and their association with mental disorders.

## Methods

A cross-sectional survey design was used with multi-state random sampling with stratification by region and displacement status. A total sample size of 3600 men and women aged 18 years and over was determined to meet the statistical requirements of the overall study. The study was limited to adults as the types of stress, mental disorders and coping strategies were expected to be very different for children. This consisted of 1200 respondents from each of the three main conflict-affected populations in Georgia: those displaced as a result of conflicts in the 1990s (‘1990s IDPs’); those displaced after the 2008 conflict (‘2008 IDPs’); and former 2008 IDPs who have returned to their home areas on the Georgian side of the border with South Ossetia after being displaced due to the 2008 conflict (‘returnees’). The study did not include returnees from the 1990s conflict as they were much harder to identify for sampling purposes.

Primary sampling units (*n* = 360; 120 per population group) were selected based on probability proportion to size method using a sampling frame of formal and informal IDP settlement population sizes throughout Georgia. Population lists were provided by the Ministry of IDPS and lists of villages in the border region with South Ossetia provided by the Governor's office in Shida Kartli region. Within each primary sampling unit, the random walk method was used to randomly select households. Within the selected household one person was randomly selected to be interviewed (based on the nearest birthday sampling technique). If there was no response at the household after three visits the next household on the route was visited.

The questionnaire was designed to capture the demographic and socio-economic status of the respondents, including their exposure to trauma, selection of coping strategies and symptoms of post-traumatic stress disorder (PTSD), anxiety and depression. The questionnaire was administered by trained fieldworkers who spoke fluent Georgian and conducted face-to-face interviews in the respondent's homes. All participants provided informed consent and data collected was confidential and anonymous. Exclusion criteria included those likely to be under the influence of alcohol or drugs, those with severe intellectual or mental impairment based on criteria describing their level of understanding, expression, behaviour and communication. Data collection took place between October and December 2011. Ethical approval was provided by the National Council on Bioethics in Georgia and the Ethics Committee of the London School of Hygiene and Tropical Medicine.

### Measures

An amended version of the Brief Coping Inventory was used to assess coping strategies (Carver, 1997). This is based on the original Coping Inventory (Carver *et al.* 1989), but reduced to 28 items addressing the same 14 coping activities in order to reduce respondent burden. In order to further reduce respondent burden, we selected only one item (rather than two) for each of the 14 coping activities. This selection process was based on discussion with local experts on which item was considered most appropriate given the study context and population and then piloting with selected IDPs to explore item meaning and clarity. An item on gambling was also added as a coping activity as it was considered relevant in the study setting based on discussions with the experts. This gave a total of 15 items/activities. The tool asks the respondent to indicate whether they generally engage in the particular coping activity. Response options are: ‘I don't usually do this at all’, ‘I usually do this a little bit’, ‘I usually do this a medium amount’, or ‘I usually do this a lot’. The wording used for the items is provided in Table 1. The instrument does not produce overall scores, but is instead designed to provide information on engagement in different types of coping (Carver *et al.* 1989; Carver, 1997).

**Table 1.** Coping items

Coping type	Item
Behavioural disengagement	I've been giving up trying to cope and deal with it
Denial	I've been refusing to believe that it has happened
Focusing on venting emotions	I've been expressing my negative feelings
Gambling	I've been gambling to help me get through it
Mental disengagement	I've been doing something to think about it less, such as work, working on my land, watching TV/films, reading, daydreaming, sleeping, or shopping
Substance use	I've been using alcohol or other drugs to help me get through it
Self-blame	I've been blaming myself for things that happened
Acceptance	I have been learning to live with it/getting used to it
Active coping	I've been taking action to try to make the situation better
Humour	I've been using humour about the situation
Positive reinterpretation and growth	I've been looking for something good in what is happening
Planning	I've been thinking hard about what steps to take
Religious coping	I've been trying to find comfort in my religion or spiritual beliefs
Use of emotional support	I've been getting emotional support and empathy from others
Use of instrumental social support	I've been getting help and advice from other people

Note: Response options of: (i) I haven't been doing this at all; (ii) I've been doing this a little bit; (iii) I've been doing this a medium amount; (iv) I've been doing this a lot.

Symptoms of PTSD were measured using the Trauma Screening Questionnaire (TSQ) which consists of ten items on PTSD symptoms over the past 1 week, with *No* (=0) and *Yes* (=1) responses which are summed to produce an overall score range of 0–10, with the TSQ's cut-off of >5 used to indicate possible PTSD (Brewin *et al.* 2002; Walters *et al.* 2007). Current symptoms of depression was measured using the Patient Health Questionnaire (PHQ-9) which consists of nine questions on depression symptoms over the last 2 weeks, with responses of: *not at all* (=0), *several days* (=1), *more than half the days* (=2) and *nearly every day* (=3), with item scores summed to produce a total score range of 0–27, with the PHQ-9's suggested cut-off of ≥10 used to indicate at least moderate depression (Kroenke *et al.* 2001). Current symptoms of anxiety were measured using the Generalised Anxiety Disorder (GAD-7) instrument which consists of seven questions on anxiety symptoms over the last 2 weeks, with the same response options and scoring as the PHQ-9, producing a total score range of 0–21, with the GAD-7's suggested cut-off of ≥10 used to indicate at least moderate anxiety (Spitzer *et al.* 2006). These instruments showed good validity and reliability with the study population, with details provided elsewhere (Makhashvili *et al.* 2014). Exposure to a range of violent and traumatic events was assessed using an adapted version of the Harvard Trauma Questionnaire (Mollica *et al.* 1992). A range of demographic and socio-economic data was also collected (e.g., gender, age, education status, marital status, household economic status).

The study instruments were translated using standard procedures involving: (i) translation from English into Georgian using professional translators, with translations reviewed by Georgian mental health experts individually and then as a group for cultural relevance, content and concept consistency, clarity and understanding; (ii) back-translation to check for accuracy, consistency and equivalence, with adjustments made accordingly; and (iii) piloting and field testing to refine the instruments further.

### Statistical analysis

Data were analysed separately for men and women given known differences in mental health outcomes, including conflict-affected persons in Georgia (Makhashvili *et al.* 2014) and also different coping types between men and women (Etzion & Pines, 1981; Billings & Moos, 1984; Stone & Neale, 1984; Norcross *et al.* 1986; Carver *et al.* 1989; Endler & Parker, 1990*a*; Greenglass, 1993, 2002; Hobfoll *et al.* 1994; Seguin & Roberts, 2015). Preliminary analysis also suggested that the significant differences were found by gender, rather than other categories such as displacement status. Descriptive analysis of demographic characteristics, mental health status and coping strategies was conducted, with differences between males and females assessed by un-paired *t*-tests.

Regression analysis was then conducted (separately for men and women) to examine associations between coping and mental health outcomes of PTSD, depression, anxiety and ≥1 mental health outcome. A bivariate regression analysis was initially conducted on associations of individual coping items with mental health outcomes. Coping items with significant (*p* < 0.05) associations were then entered into a multivariate regression to produce adjusted Odds Ratios (ORs) between the individual coping strategies and mental health outcomes (with separate multivariate models run for each mental health outcome). Additional demographic and socio-economic items were included in the regression model to adjust for potential confounding, based on those that showed a significant association in bivariate analysis. These variables were age, displacement status, exposure to multiple traumatic events, marriage status, employment status and access to social support.

Co-linearity between the individual coping strategies and mental health outcomes was assessed using correlation analysis, none of which reached a level considered to be highly correlated (*r* = ≥0.80) (which is to be expected as the tool seeks to measure a range of coping strategies rather than a single dimension of coping). The ability of the Brief Coping Inventory to measure relatively distinct aspects of coping has been reported elsewhere, in addition to showing convergent and discriminate validity (Carver *et al.* 1989; Carver, 1997). The data were weighted to reflect the actual proportions of 1990s IDPs (9.3%), 2008 IDPs (57.1%) and returnees (33.6%) in the overall conflict-affected populations of Georgia. The analysis was adjusted for the cluster design. Statistical significance was taken to be *p* < 0.05. STATA version 9.2 (Stata Corporation, College Park, Texas, USA) was used for the analysis.

## Results

### Sample characteristics, exposure to trauma and mental health status

In total 3600 interviews were conducted and the overall response rate was 84%. Table 2 shows the majority of the sample were female (65.3%) which reflects trends of men leaving Georgia to find employment elsewhere (Rytwinski *et al.* 2013). The majority of respondents were married (59.0%), with the rest either widowed (19.4%), single (17.1%) or divorced/separated (4.5%). Almost all respondents had completed secondary school (69.6%) or higher education (21.1%). Rates of unemployment were high, with 33.7% of men and 21.7% of women reporting being unemployed. 54.1% of respondents reported having a very bad household economic status. The majority (80.2%) of the respondents had experienced one or more trauma-related events. More men than women were exposed to multiple traumatic events and reported higher levels of direct exposure to combat and lack of shelter (*p* < 0.001).

**Table 2.** Sample characteristics

	Men (*n* = 1248)			Women (*n* = 2352)		Total (*n* = 3600)	
Characteristics	*N*	(%)		*N*	(%)	*N*	(%)
Age							
18–39 years	458	(36.69)	0	790	(33.57)	1248	(34.7)
40–59 years	440	(35.31)	0	814	(34.59)	1254	(34.8)
60+ years	349	(28)	0	749	(31.84)	1098	(30.5)
Education level							
Completed higher education	253	(20.27)		507	(21.57)	760	(21.1)
Completed secondary school	872	(69.87)		1633	(69.48)	2505	(69.6)
Primary/incomplete secondary	123	(9.86)		210	(8.95)	333	(9.3)
Marital status							
Married	816	(65.38)		1307	(55.64)***	2123	(59.02)
Single	315	(25.25)		301	(12.83)***	616	(17.13)
Divorced/separated	32	(2.56)		129	(5.48)**	161	(4.48)
Widowed	85	(6.81)		612	(26.05)****	697	(19.37)
Employment status							
Unemployed	420	(33.7)		511	(21.7)***	931	(25.9)
Not employed and not seeking work	120	(9.7)		234	(10.0)	354	(9.8)
In full-time regular work	283	(22.8)		316	(13.4)***	599	(16.7)
In irregular paid work	58	(4.7)		35	(1.5)***	93	(2.6)
Self-employed	60	(4.8)		74	(3.2)	134	(3.7)
Housewife				446	(19.0)***	446	(12.4)
Retired	277	(22.2)		686	(29.2)***	963	(26.8)
Other	27	(2.1)		47	(2.0)	74	(2.1)
Household economic status							
Very good	7	(0.59)		5	(0.21)	12	(0.33)
Good	23	(1.86)		33	(1.39)	56	(1.56)
Average	551	(44.19)		1032	(43.89)	1583	(43.98)
Bad	460	(36.85)		850	(36.15)	1310	(36.40)
Very bad	206	(16.5)		432	(18.37)	638	(17.73)
Trauma exposure							
Lack of shelter	651	(52.2)		918	(39.0)***	1569	(30.6)
Serious injury	309	(24.7)		334	(14.2)***	643	(12.5)
Directly in combat situation	635	(50.9)		993	(42.2)***	1628	(31.7)
Physical abuse	29	(2.3)		67	(2.9)	96	(1.9)
Sexual abuse	5	(0.4)		1	(0.1)	6	(0.1)
Being abducted	32	(2.6)		18	(0.8)***	50	(1.0)
Been tortured	36	(2.9)		17	(0.7)*	53	(1.0)
Experienced murder, violence acts against family/friends	310	(24.8)		514	(21.9)*	824	(16.1)
Witnessed murder, violence acts against stranger	129	(10.4)		132	(5.6) ***	261	(5.1)
Multiple traumatic events							
No events	195	(15.6)		518	(22.0)***	713	(19.8)
1 event	301	(24.1)		590	(25.1)	891	(24.8)
2 events	254	(20.4)		520	(22.1)	774	(21.5)
3+ events	498	(39.9)		724	(30.8)***	1222	(33.9)

% adjusted for weightings in the total sample and adjusted for cluster survey study design.

* = *p* < 0.05, ** = *p* < 0.01, *** = *p* < 0.001 significant difference in % of men *v*. women.

The prevalence of mental health problems are given in Table 3. Symptoms suggestive of PTSD were reported by 23.6% of the population, and symptoms suggestive of moderate to severe of depression and anxiety were reported by 14.4 and 10.9%, respectively. Overall, 30.5% of the population reported symptoms suggestive of any mental health problem (i.e., one or more the disorders). The prevalence of mental health problems (all reported types) was significantly higher in women than men.

**Table 3.** Frequency of different coping activities and prevalence of mental problems

	Men		Women		Total	
	*N*	(%)	*N*	(%)	*N*	(%)
Coping strategy used						
Behavioural disengagement	377	(15.9)	195	(16.6)	572	(16.6)
Denial	258	(22.3)	575	(26.8)**	833	(28.4)
Focus on and venting of emotions	340	(27.6)	574	(24.7)	914	(24.7)
Gambling	73	(5.9)	67	(2.9)**	140	(3.7)
Mental disengagement	729	(59.4)	1456	(62.9)*	2185	(63.2)
Substance abuse	90	(7.2)	126	(5.4)*	216	(5.7)
Self-blame	199	(16.9)	416	(18.9)	615	(18.6)
Acceptance	816	(67.2)	1537	(67.8)	2353	(69.2)
Active coping	790	(64.7)	1385	(60.5)*	2175	(66.6)
Humour	247	(20.6)	383	(17.1)*	630	(17.3)
Positive reinterpretation and growth	448	(37.6)	851	(38.7)	1299	(41.9)
Planning	811	(65.8)	1497	(65.0)	2308	(70.9)
Religion	431	(36.2)	1141	(50.8)***	1572	(45.6)
Use of emotional social support	748	(60.6)	1427	(61.7)	2176	(63.7)
Use of instrumental social support	771	(62.4)	1606	(69.3)**	2377	(69.7)
Mental health problems						
Post-traumatic stress disorder symptoms	235	(19.2)	612	(26.4)***	847	(23.6)
Depression symptoms	142	(11.4)	377	(16.0)**	519	(14.4)
Anxiety symptoms	96	(7.7)	299	(12.7)**	395	(10.9)
Any mental health disorder symptoms	332	(25.3)	764	(31.8)***	1096	(30.5)

Total number excludes those who answered ‘don't know’ or ‘refused to answer’.

% adjusted for weightings in the total sample and for cluster survey study design.

* = *p* < 0.05, ** = *p* < 0.01, *** = *p* < 0.001 in % of men *v*. women.

Items based on adapted Brief Coping Inventory measure. For wording of individual items, see Table 1.

### Coping strategies

The self-reported coping strategy use is collated in Table 3. The most commonly reported coping strategies were planning, acceptance, use of instrumental and emotional social support, active coping and mental disengagement. The least widely reported were gambling and substance abuse. The reported use of coping strategies varied significantly between men and women for 8 of the 15 coping strategies (*p* < 0.05 to <0.001).

### Association between coping strategy and mental health status

Table 4 shows the associations between coping strategies and mental health outcomes. The coping strategies significantly associated with PTSD symptoms included: behavioural disengagement for men and women; denial for men and women; and gambling for men and women; whereas in women the use of humour and emotional social support were associated with less PTSD symptomatology, but no such associations were observed for men.

**Table 4.** Association between coping and mental health outcomes

	PTSD				Depression				Anxiety				Any mental health condition (≥1 condition)			
	Males		Females		Males		Females		Males		Females		Males		Females	
Coping strategy	OR	[95% CI]	OR	[95% CI]	OR	[95% CI]	OR	[95% CI]	OR	[95% CI]	OR	[95% CI]	OR	[95% CI]	OR	[95% CI]
Behavioural disengagement	1.66	[1.12–2.48]*	1.53	[1.11–2.13]*	2.22	[1.30–3.79]*	1.91	[1.34–2.72]**	2.14	[1.31–3.47]**	2.21	[1.54–3.18]**	1.69	[1.17–1.63]**	1.84	[1.34–2.51]**
Denial	1.65	[1.11–2.44]*	1.98	[1.47–2.56]**	1.81	[1.10–2.95]*	1.91	[1.39–2.63]**			1.57	[1.07–2.30]*	1.30	[0.90–1.90]	2.03	[1.53–2.69]**
Focus on venting emotions	1.16	[0.82–1.63]			1.71	[1.08–2.69]*	1.68	[1.25–2.28]**	1.31	[0.80–2.14]	1.55	[1.10–2.20]*	1.18	[0.86–1.63]	1.26	[0.98–1.62]
Mental disengagement			1.09	[0.83–1.44]							1.55	[1.05–2.30]*			1.28	[0.98–1.68]
Substance abuse					2.99	[1.49–5.90]**	1.84	[1.14–2.96]*	2.81	[1.53–5.12]**	1.65	[1.01–3.11]*	2.63	[1.55–4.48]**	1.91	[1.25 –2.93]**
Gambling	1.88	[1.07–3.29]*	1.58	[1.01–2.45]*												
Humour			0.66	[0.46–0.95]*							0.35	[0.20–0.62]**			0.63	[0.45 –0.89]**
Use of emotional social support			0.47	[0.35–0.63]**							0.63	[0.45–0.89]**			0.56	[0.42 –0.73]**
Use of instrumental social support			1.28	[0.92–1.78]												
Active coping					0.55	[0.33–0.93]*	0.65	[0.46–0.93]*			0.69	[0.47–1.02]				
Acceptance			1.33	[1.00–1.76]											1.36	[1.05–1.77]*
Religion															0.65	[0.49–0.84]**

* = *p* < 0.05, ** = *p* < 0.01. CI, confidence interval; OR, odds ratio (adjusted); NS, not significant (*p* > 0.05). Multivariate regression analysis using significant (*p* < 0.05) coping strategies and other significant factors (age, displacement status, marital status, trauma exposure and social support) identified in initial bivariate analysis. Separate multivariate regression models run for each mental health outcome. Empty cells are for variables which were not significant in the initial bivariate analysis. Coping strategy items based on adapted Brief Coping Inventory measure. For wording of individual items, see Table 1.

The coping strategies significantly associated with symptoms of depression included: behavioural disengagement for men and women; denial for men and women; and substance abuse for men and women; whereas active coping was associated with less depression in men and women.

The coping strategies significantly associated with GAD among women were behavioural disengagement, denial, focus on venting emotions, mental disengagement and substance abuse; while humour and emotional social support were significantly associated with being protective against GAD. Among men, only behavioural disengagement and substance abuse were significantly associated with symptoms of generalised anxiety.

For any mental health condition, behavioural disengagement and substance use showed significant associations among men. Among women, behavioural disengagement, denial and substance use were significantly associated with any mental health condition; whereas humour, use of emotional support, acceptance and religion were all significantly associated with a lower probability of any mental health condition (Table 4).

## Discussion

This is one of the few studies to examine coping strategies and their association with mental health among conflict-affected civilian populations in a low- and middle-income setting (Seguin & Roberts, 2015). The variance between men and women in coping strategies employed reflects findings from elsewhere (Etzion & Pines, 1981; Billings & Moos, 1984; Stone & Neale, 1984; Norcross *et al.* 1986; Carver *et al.* 1989; Endler & Parker, 1990*a*; Greenglass, 1993, 2002; Hobfoll *et al.* 1994; Seguin & Roberts, 2015). Explanations for these commonly relate to the influence of context. For example, Folkman and Lazarus observed a strong link between the work context and problem-focused coping and reasoned that the observed gendered differences in problem-based coping may result from gender-based differences in jobs, rather than a general disposition, with men more likely to be in positions which provide opportunities to engage in problem-solving behaviours (Folkman & Lazarus, 1980). Hobfoll *et al*. contend that women are more likely than men to encounter stressful situations that offer little possibility of control, and may lead the former to invoke emotion-focused coping over problem-focused (Hobfoll *et al.* 1994).

Denial and behavioural disengagement showed some of the strongest associations with poor mental health in men and women. Denial is a somewhat controversial coping strategy. It can be considered useful, minimising distress and thereby facilitate dealing with the current situation (Cohen & Lazarus, 1973; Wilson, 1981). Alternatively, it can create additional difficulties since it impedes addressing the problem, thereby making it even more difficult to manage the emotional distress associated with the situation (Matthews *et al.* 1983). A third view is that denial may be useful during the early stages of a stressful event but impede recovery later on (Levine *et al.* 1987). The use of denial and behavioural disengagement by our study population reflects the aetiology of PTSD, since it is associated with incomplete processing of a traumatic event and therefore may aggravate these forms of coping (Ehlers & Clark, 2000). Therefore, while denial can be helpful, it may become avoidance which is unhelpful and, indeed, pathological as demonstrated by the criteria of avoidance for PTSD (American Psychiatric Association, 2013). Other studies with conflict-affected populations suggest that activities related to disengagement and denial are generally, but by no means universally, associated with poorer mental health outcomes (Seguin & Roberts, 2015). Disengaging from the trauma has largely been shown to impede recovery (Aldwin & Revenson, 1987; Carver *et al.* 1989). Given this and the requirement for avoidance to be present for a diagnosis of PTSD, it is no surprise that trauma focused psychological treatments have been found to be superior in efficacy to non-trauma focused ones (Bisson *et al.* 2013) and are recommended as the first line treatment for PTSD by the National Institute for Health and Care and Excellence (National Collaborating Centre for Mental Health, 2005) and the World Health Organisation (Tol *et al*. 2013).

Although infrequently reported, substance abuse was the coping strategy most strongly associated with mental health problems (with the exception of PTSD) in both men and women, with the association almost twice as strong for men than women. Alcohol is frequently used to avoid confronting painful memories and commonly co-morbid with PTSD and other mental disorders (Kessler *et al.* 1995). Alcohol consumption in this study population has been reported elsewhere, with nearly a third of men who consumed alcohol classified with hazardous alcohol use, whereas for women it was 2% (Roberts *et al.* 2014). This is consistent with other studies showing that typically more men than women turn to alcohol to deal with stress (Carver *et al.* 1989; Weaver & Roberts, 2010; Ezard, 2012).

The use of active coping, which can be defined as the process of taking active steps to try to remove or circumvent the stressor or to ameliorate its effects (Carver *et al.* 1989), was common in our study and findings suggest it was protective against depression for both men and women. Use of active coping has been reported in other conflict-affected populations and largely shown to be supportive of mental health (Seguin & Roberts, 2015).

Religion was also associated with a lower probability of having a mental health condition in women. Engaging in religious activities and receiving advice from religious groups have previously been shown to be protective against poor mental health in other conflict-affected populations (Ruwanpura *et al.* 2006; Hardgrove, 2009; Sousa, 2013; Seguin & Roberts, 2015).

Emotional social support was frequently reported among men and women, but only associated with better mental health outcomes among women, as was use of humour. As noted above, the use and benefit of emotional support among women may reflect the context and types of stressful situations encountered by women that may lead them to benefit more from emotion-focused coping strategies (Hobfoll *et al.* 1994). Perceived good social support following traumatic events was found to be one of the strongest factors associated with reduced rates of PTSD in two large meta-analyses (Brewin *et al.* 2000; Ozer *et al.* 2003).

While there have been a number of studies measuring the effectiveness of interventions for mental health outcomes among conflict-affected populations, there are very few studies that have been conducted on the effectiveness of coping-related interventions (Tol *et al.* 2011; Blanchet *et al.* 2015; Seguin & Roberts, 2015). Future studies in Georgia and other conflict-affected and post-conflict settings should seek to explore effective interventions to support and strengthen coping strategies among conflict-affected populations. These should be community-driven initiatives to support active coping, support-seeking, positive cognitive restructuring and problem-solving, which have been associated with better mental health in Georgia and other conflict-affected populations (Seguin & Roberts, 2015). Furthermore, the interventions must address negative coping strategies, such as disengagement and substance abuse which can be associated with poorer mental health (Seguin & Roberts, 2015). These could be led by community-based organisations in the affected communities, with support from non-governmental organisations, faith-based organisations and governmental agencies with expertise in mental health and psychosocial support and in line with humanitarian guidelines (IASC, 2007). The interventions and their evaluations should also be gender sensitive given the different coping responses observed between men and women.

### Limitations

Due to the cross-sectional nature of the study, the relationship between coping strategies and symptoms of common mental disorders as PTSD, depression or anxiety cannot be considered causal. Nevertheless, the aetiology of PTSD suggests that mental health problems develop first and lead individuals to choose a coping strategy that either helps or exacerbates the problem (Ehlers & Clark, 2000). As acknowledged by its authors, the Brief Coping Inventory may not capture all coping strategies due to the diversity of coping behaviours amongst different population groups, especially given the challenge of achieving local social and cultural relevance and appropriateness (Carver *et al.* 1989). In addition, we used an adapted version of the Brief Coping Inventory which relied on single items for each coping activities, rather than dual items used in the original version. This may have affected reporting of the different coping strategies by respondents. However, we did select the questions based on expert consensus and piloting, although it was not formally validated with the study population. The mental health measures used in this study were not developed specifically for the Georgian population, but they were validated in a pilot study with IDPs and the psychometric properties of the instruments with the study populations are reported elsewhere (Makhashvili *et al.* 2014). Stigma associated with mental health problems and certain coping strategies (e.g., substance misuse and gambling), and the time interval since traumatic events occurred, may have led to reporting bias. The close relationship between some of the phenomena associated with common mental disorders (such as poor concentration and dysphoria) with some of the coping strategies could potentially be attributable to them measuring the same latent construct. However, the different nature of the questions (i.e., activities for coping *v.* feelings/symptoms associated with a disorder) suggests this was not the case and the Brief Coping Inventory has also demonstrated discriminate validity (Carver *et al.* 1989; Carver, 1997). Finally, the study excluded respondents who were under the influence of alcohol or drugs. This could potentially have caused a bias as the use of alcohol and drugs can be considered as forms of coping. We mitigated this risk of bias by returning to respondent homes again to interview them when they were not considered to be under the influence of alcohol and drugs. Less than ten respondents were excluded because of this reason and so potential bias is considered extremely low.

### Conclusions

This study with conflict-affected persons in Georgia identified a number of coping strategies associated with better and poorer mental health. These findings may be useful to Georgian authorities, NGOs and community organisations who work with the conflict-affected persons to design interventions that support coping strategies most strongly associated with good mental health. Such strategies may be particularly pertinent given the barriers faced by conflict-affected persons in using mental health services in Georgia (Chikovani *et al.* 2015). Such interventions should be culturally appropriate, gender-sensitive, effective and feasible.
